# Loss of *Dab1* Alters Expression Patterns of Endocytic and Signaling Molecules During Embryonic Lung Development in Mice

**DOI:** 10.3390/life15091395

**Published:** 2025-09-03

**Authors:** Petar Todorović, Mirko Maglica, Nela Kelam, Natalija Filipović, Azer Rizikalo, Ilija Perutina, Josip Mišković, Yu Katsuyama, Katarina Vukojević

**Affiliations:** 1 Department of Anatomy, Histology and Embryology, University of Split School of Medicine, 21000 Split, Croatia; petar.todorovic@mefst.hr (P.T.); nela.kelam@mefst.hr (N.K.); natalija.filipovic@mefst.hr (N.F.); 2 Department of Anatomy, School of Medicine, University of Mostar, 88000 Mostar, Bosnia and Herzegovina; mirko.maglica@mef.sum.ba (M.M.); azer.rizikalo@mef.sum.ba (A.R.); ilija.perutina@mef.sum.ba (I.P.); josip.miskovic@mef.sum.ba (J.M.); 3 Anatomy Department of Anatomy, Shiga University of Medical Science, Otsu 520-2192, Japan; kats@belle.shiga-med.ac.jp; 4 Mediterranean Institute for Life Sciences, University of Split, 21000 Split, Croatia; 5 Center for Translational Research in Biomedicine, University of Split School of Medicine, 21000 Split, Croatia

**Keywords:** Megalin, Cubilin, Caveolin-1, GIPC1, Dab2IP, *yotari*, endocytosis, lung injury

## Abstract

Lung development is governed by tightly regulated signaling mechanisms, including endocytosis-mediated pathways critical for epithelial–mesenchymal communication and tissue remodeling. This study investigated the effects of *Dab1* deficiency on the expression of endocytic and signaling-related proteins, Megalin, Cubilin, Caveolin-1, GIPC1, and Dab2IP, during embryonic lung development in *yotari* mice. Using immunofluorescence and quantitative image analysis, protein expressions were compared between *yotari* and wild-type embryos at gestational days E13.5 and E15.5. Results showed significantly reduced expression of Caveolin-1 in the *yotari* epithelium across both stages, along with diminished mesenchymal levels of Megalin and GIPC1 at E13.5. Cubilin and Dab2IP expression patterns showed no statistically significant differences, although developmental and compartmental shifts were observed. These findings suggest that *Dab1* deficiency selectively disrupts endocytic and signaling scaffolds crucial for branching morphogenesis and alveolar maturation. The altered spatiotemporal expression of these proteins underscores the essential role of *Dab1* in regulating lung epithelial–mesenchymal dynamics and maintaining developmental homeostasis during critical stages of organogenesis.

## 1. Introduction

Lung development is a tightly coordinated process regulated by epithelial–mesenchymal signaling and transcriptional networks. It proceeds through distinct stages, embryonic, pseudoglandular, canalicular, saccular, and alveolar, resulting in a specialized gas-exchange organ [1,2]. Key pathways, including Wnt, FGF, BMP, and SHH, orchestrate progenitor specification, branching morphogenesis, and alveolar differentiation, while developmental mechanisms also inform regenerative strategies for congenital and acquired lung disease [1,2]. Central to this developmental program are apoptosis and endocytosis, which serve as critical regulators of tissue homeostasis and morphogenetic remodeling [3,4]. Regulated cell death, including apoptosis, necroptosis, pyroptosis, and autophagy-associated death, plays a fundamental role in lung development by eliminating redundant cells, maintaining tissue homeostasis, and shaping alveolar architecture [5]. At the same time, endocytosis mediates the internalization of receptors and the regulation of signaling [3], which are essential for epithelial–mesenchymal communication [6].

*Yotari* mutant mice, which spontaneously acquire a mutation in the *Dab1* gene, exhibit histological abnormalities in the central nervous system, remarkably similar to those observed in *reeler* (*reelin^−/−^*) mice, suggesting that Dab1 and Reelin function within the same signaling pathway [7,8]. Although research on *yotari* mice has primarily focused on the nervous system, Dab1 signaling also modulates peripheral organogenesis [9,10,11]. Recent work has shown that silencing *Dab1* alters autophagy markers and epithelial–mesenchymal interactions during embryonic lung development at E13.5 and E15.5 [12].

Megalin (LRP-2/GP330), a member of the low-density lipoprotein receptor family, is an endocytic receptor mainly expressed in polarized epithelial cells (type II pneumocytes), but there is increasing recognition of its involvement in lung embryonic development [13]. *Megalin* knockout mice die perinatally due to respiratory insufficiency, characterized by abnormal epithelial tissues, including those in the lungs [13,14]. The receptor’s expression begins early in lung development and persists through gestation, suggesting a fundamental role in lung morphogenesis [13].

Cubilin serves as a peripheral membrane co-receptor that forms functional complexes with Megalin to facilitate ligand internalization [14,15]. This interaction is crucial for the endocytosis of many substrates via the Megalin-dependent pathway in epithelial tissues, including the lungs [14]. The Megalin–Cubilin complex mediates the uptake of transferrin, albumin, and surfactant precursors, processes that are crucial for lung development and function [16]. Cubilin’s expression pattern during lung development suggests it plays a role in epithelial differentiation and maturation [13,16].

Caveolin-1 (Cav-1) is the primary structural protein of caveolae, membrane invaginations involved in signaling, endocytosis, and transcytosis. Cav-1 regulates key signaling pathways [17,18], including eNOS, the TGF-β, MAPK/ERK, and eNOS pathways, which are essential for lung morphogenesis and homeostasis [19,20]. *Cav-1* knockout mice exhibit delayed alveolar development, characterized by wall thickening, hypercellularity, and fibrotic remodeling [18,20]. Mechanical stretch, an essential developmental signal, drives translocation of Cav-1 from the plasma membrane to the cytoplasm in fetal type II alveolar epithelial cells, underscoring its role as a mechanosensitive regulator during lung development [19].

GIPC1 (GAIP-interacting protein, C-terminus) is a post-synaptic density protein 95/disks large/zonula occludens-1 domain adaptor that links transmembrane receptors to endocytic and recycling pathways, including myosin VI–driven trafficking [21,22]. Recent studies have highlighted broader functions of GIPC1 beyond its expression profile. GIPC1 stabilizes the scavenger receptor SR-B1, thereby enhancing cholesterol uptake and lipid homeostasis [21]. Moreover, GIPC proteins modulate PlexinD1 signaling during vascular and branching morphogenesis, underscoring their developmental importance [23]. GIPC1 regulates signaling pathways, such as the PDGFR/PI3K/AKT pathway, and the receptor recycling of TGFβR3 and α5β1-integrin—processes crucial for branching morphogenesis and alveolarization [22,24].

Dab2IP (Disabled-2 Interacting Protein) is a RAS-GTPase-activating protein that functions as a cytoplasmic scaffold protein coordinating multiple signaling pathways essential for cellular homeostasis [25,26]. In lung biology, Dab2IP expression is significantly downregulated by cigarette smoke through epigenetic mechanisms involving EZH2-mediated H3K27me3 histone modifications [27,28]. Notably, Dab2IP belongs to the Disabled protein family, alongside Dab1, which shares structural similarities and serves as a critical adaptor protein in cellular signaling [29,30].

We hypothesize that the combined dysregulation of Megalin, Cubilin, Caveolin-1, GIPC1, and Dab2IP in *yotari* lungs disrupts endocytosis-linked checkpoints, suppresses apoptosis, and ultimately remodels branching patterns and alveolar maturation. The spatiotemporal expression of these proteins across critical developmental stages E13.5 and E15.5 will clarify how endocytic scaffolds and death-signaling adaptors cooperate to safeguard normal pulmonary organogenesis. Therefore, to validate this hypothesis, in the present study, we analyzed the expression patterns of these proteins in developing *yotari* and control lungs using immunohistochemistry and quantitative evaluation.

## 2. Materials and Methods

### 2.1. Ethics

The Shiga University of Medical Science Guidelines for the Care and Use of Laboratory Animals permitted the use of animals. The University of Split School of Medicine’s Ethical Committee gave its approval to the study, which was carried out in accordance with Directive 2010/63/EU on the protection of animals used for scientific purposes (protocol code no. 2181-198-03-04-23-0073; 27 September 2023).

### 2.2. Sample Collection

This study utilized homozygous *yotari* (*yot*) mice and wild-type (wt) C57BL/6N mice, both of which were genetically equivalent to their respective strains [31]. Animals were housed in groups of three or four per standard polycarbonate cage, maintained at a controlled room temperature of 23 ± 2 °C, and provided with unlimited access to regular chow and water. The light–dark cycle was kept on a 12-h schedule. At each timepoint, three mice from both genotypes (*yotari* and wild-type) were included. Pregnant females were euthanized on gestational days E13.5 and E15.5. Their embryos were fixed by immersion in 4% paraformaldehyde (PFA) in phosphate-buffered saline (PBS). For genotyping, DNA was extracted, and PCR was conducted using the following primers:

*yotari:* GCCCTTCAG-CATCACCATGCT and CAGTGAGTACATATTGTGTGAGTTCC;

wild-type *Dab1* locus: GCCCTTCAGCATCACCATGCT and CCTTGTTTCTTTGCTTTAAGGCTGT [32].

### 2.3. Immunofluorescence Staining

After fixation, tissues were embedded in paraffin and sectioned at a thickness of 5 µm. Sections were placed on glass slides, deparaffinized in xylene, and rehydrated through a graded ethanol series to distill water. Antigen retrieval was performed by heating the sections in sodium citrate buffer (pH 6.0) at 95 °C for 30 min using a water steamer. To minimize nonspecific binding, slides were treated with blocking buffer (ab64226, Abcam, Cambridge, UK) for 30 min. Primary antibodies (Table 1) were then applied and incubated overnight in a humidified chamber. The following day, sections were washed in PBS and exposed to the appropriate secondary antibodies (Table 1) for 1 h. Finally, after PBS rinses, slides were mounted with Immu-Mount medium (Thermo Shandon, Pittsburgh, PA, USA). Antibody specificity was verified by a pre-adsorption control, in which each primary antibody was mixed with its corresponding peptide antigen in blocking solution before application. No signal was detected in these controls. Likewise, omitting the primary antibody from the immunofluorescence protocol resulted in no observable staining, indicating that there was no nonspecific reactivity from the secondary antibodies.

### 2.4. Data Acquisition and Analysis

To assess protein expression levels, we quantified the percentage of fluorescent signal area from captured images. Image acquisition was performed using a fixed exposure time with a fluorescence microscope (BX51, Olympus, Tokyo, Japan) and a Nikon DS-Ri2 camera, controlled by NIS-Elements software (v5.22.00).

Image post-processing was performed using Adobe Photoshop (v21.0.2, Adobe Systems, San Jose, CA, USA) and ImageJ software (v1.53o, NIH, Bethesda, MD, USA). The background fluorescence was removed in Photoshop using the “Levels” function. The epithelial compartment was then carefully outlined using the lasso tool, cut, and pasted into a blank image of identical dimensions, effectively separating it from the underlying lamina propria.

To isolate green fluorescence, the red channel was removed from both epithelial and mesenchymal images in ImageJ. Each image was duplicated, and one copy was processed with a median filter (radius = 10) to minimize background noise. The filtered image was then subtracted from the original to extract the accurate positive signal. Thresholding was performed using the Triangle method, and the resulting images were converted to 8-bit grayscale. The “Analyze Particles” function in ImageJ was used to calculate the percentage of the area occupied by the fluorescent signal. Since not all images contained complete tissue coverage, measured area percentages were corrected for background space. Using the “Magic Wand” tool in Photoshop, the number of total pixels and non-tissue (empty) pixels was determined as described previously [11,33]. This adjusted value was then used in all statistical evaluations.

### 2.5. Statistical Analysis

All statistical tests were performed using GraphPad Prism version 9.0.0 (GraphPad Software, San Diego, CA, USA). Data were expressed as mean values ± standard deviation (SD). Two-way analysis of variance (ANOVA) followed by Tukey’s post hoc test was used to compare protein expression levels across different groups and timepoints. Differences were considered statistically significant when *p*-values were *p* < 0.05.

## 3. Results

In the lungs of wt and *yotari* mice, the immunoexpression of Megalin, Cubilin, Caveolin-1, GIPC1, and Dab2IP was observed in the embryonic respiratory epithelium and mesenchyme. Each genotype included at least four specimens at both timepoints, E13.5 and E15.5.

### 3.1. Megalin Expression

**Figure 1:**
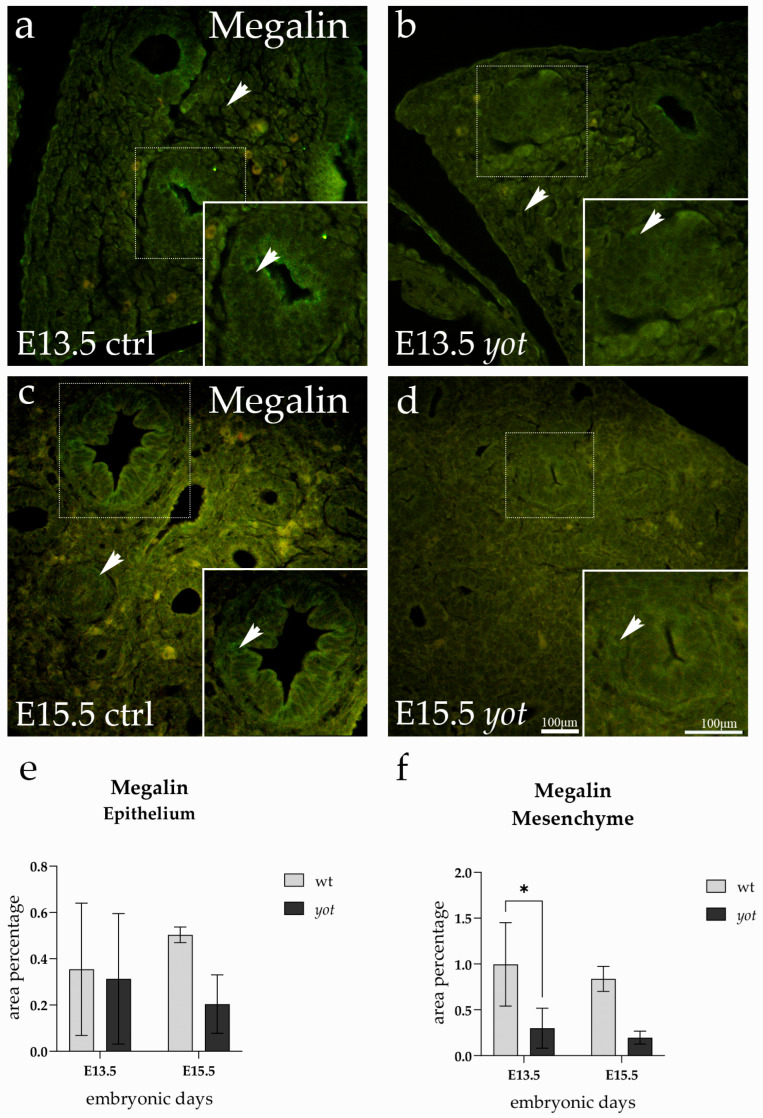
Immunofluorescence staining of Megalin in developing control (ctrl) and *yotari* (*yot*) lung tissue (**a**–**d**). Comparative expression of Megalin in the lungs at embryonic day 13.5 (E13.5) and 15.5 (E15.5) is shown in (**a**–**d**). Arrows in each substructure of the lungs indicate positive staining of Megalin. Magnification: ×40; scale bar: 100 µm. The area percentage of Megalin in control specimens (ctrl) and *yotari* mice per structure (epithelium and mesenchyme) throughout different stages of developing lungs (**e**,**f**). Two substructures were assessed at E13.5 and E15.5. Data are displayed as the mean ± SD (vertical line) and analyzed by a two-way ANOVA test followed by Tukey’s multiple comparison test. Significant differences are indicated by the following: * *p* < 0.05. The statistical details supporting these findings are provided in Supplementary Tables S1 and S2.

The signal was predominantly localized in the cytoplasm and varied in intensity across different tissue compartments and developmental stages (Figure 1).

In the epithelium, Megalin expression appeared broadly distributed in all groups. Quantification revealed no statistically significant differences between control and *yotari* lungs at E13.5. Likewise, no difference was found between control lungs at E13.5 and E15.5. Epithelial expression remained unchanged between control and *yotari* lungs at E15.5. No significant change was observed between *yotari* lungs at E13.5 and E15.5 (Figure 1e). Although these values did not differ statistically, the green signal in the epithelium was most prominent in both control and *yotari* lungs at E15.5, particularly along the airway lining (Figure 1c,d).

In the mesenchymal compartment, the strongest Megalin signal was observed in control lungs at E13.5 (Figure 1a), where numerous individual cells displayed distinct cytoplasmic staining. This signal was significantly reduced in *yotari* lungs at the same developmental stage (*p* < 0.05) (Figure 1f). No other statistically significant differences were detected among the remaining mesenchymal groups. Figure 1a highlights this difference, with an arrow indicating the prominent mesenchymal staining in control E13.5 lungs, which was quantitatively confirmed.

### 3.2. Cubilin Expression

**Figure 2:**
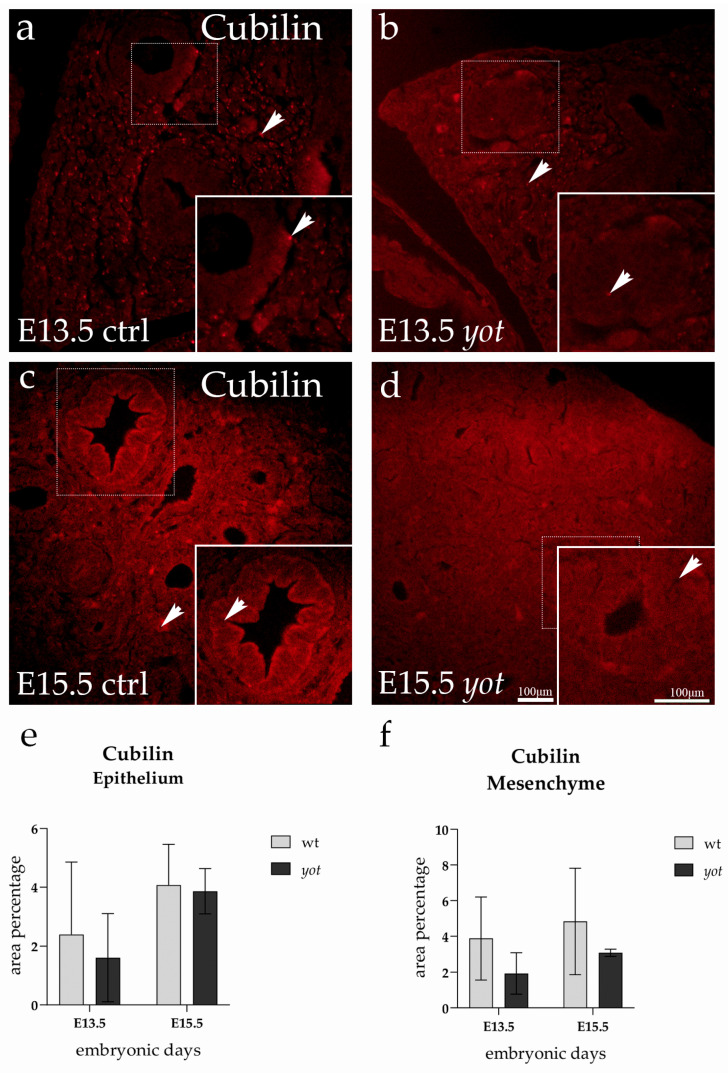
Immunofluorescence staining of Cubilin in developing control (ctrl) and *yotari* (*yot*) lung tissue (**a**–**d**). Comparative expression of Cubilin in the lungs at embryonic day 13.5 (E13.5) and 15.5 (E15.5) is shown in (**a**–**d**). Arrows in each substructure of the lungs indicate positive staining of Cubilin. Magnification: ×40; scale bar: 100 µm. The area percentage of Cubilin in control specimens (ctrl) and *yotari* mice per structure (epithelium and mesenchyme) throughout different stages of developing lungs (**e**,**f**). Two substructures were assessed at E13.5 and E15.5. Data are displayed as the mean ± SD (vertical line) and analyzed by a two-way ANOVA test followed by Tukey’s multiple comparison test. The statistical details supporting these findings are provided in Supplementary Tables S1 and S2.

Cubilin expression was detected throughout both the epithelial and mesenchymal compartments of the developing lung in control and *yotari* embryos at E13.5 and E15.5. The immunofluorescence signal was predominantly localized to the cytoplasm, with a tendency toward accentuation at the apical membrane in epithelial cells (Figure 2).

At E13.5, Cubilin was more prominently expressed in the mesenchymal tissue of control lungs, where numerous individual cells exhibited apparent cytoplasmic staining (Figure 2a). This mesenchymal signal, while visually noticeable, was not significantly different when compared to the *yotari* group. By E15.5, Cubilin expression appeared to shift; a more intense signal became evident in the epithelial layer of control lungs, particularly along the luminal surface of the developing airway structures (Figure 2c). In contrast, the corresponding epithelial regions in *yotari* lungs exhibited a more diffused and modest signal.

Despite these visible trends in signal intensity and compartmental distribution, statistical analysis revealed no significant differences in Cubilin expression between any of the groups or timepoints (Figure 2e,f). The comparisons between control and *yotari* lungs at E13.5, as well as at E15.5, showed similar overall expression patterns. Likewise, there were no changes in Cubilin expression between E13.5 and E15.5 within either genotype.

### 3.3. Caveolin Expression

**Figure 3:**
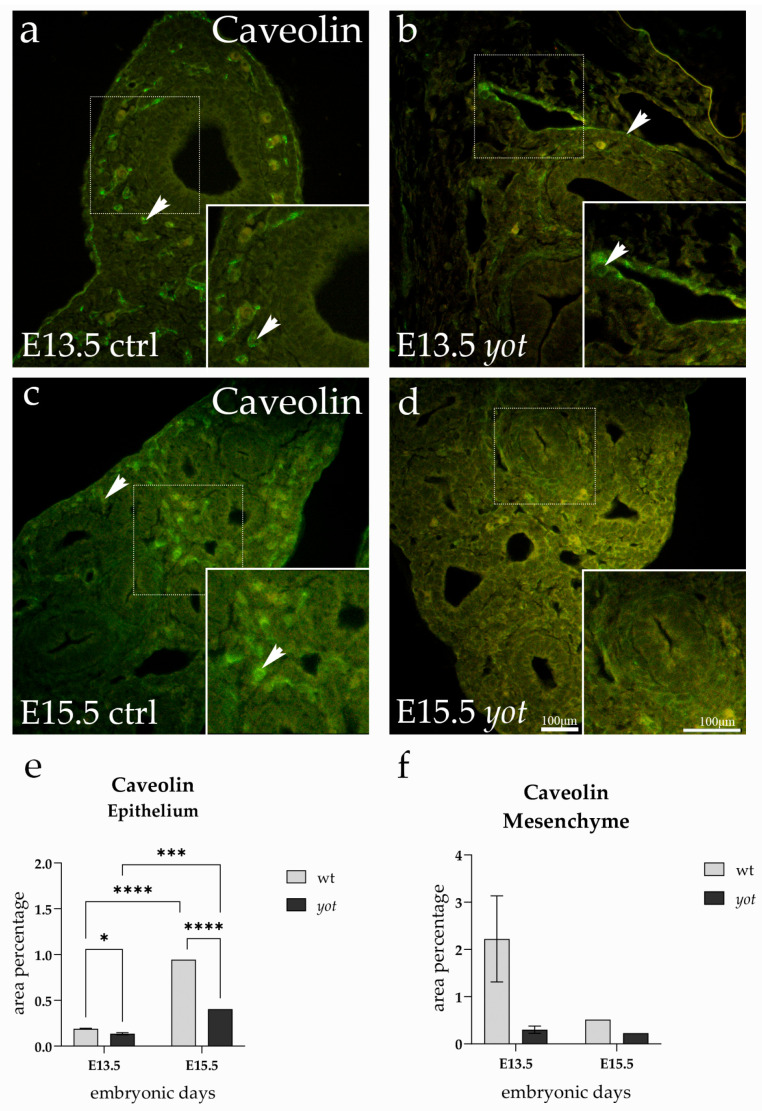
Immunofluorescence staining of Caveolin in developing control (ctrl) and *yotari*(*yot*) lung tissue (**a**–**d**). Comparative expression of Caveolin in the lungs at embryonic day 13.5 (E13.5) and 15.5 (E15.5) is shown in (**a**–**d**). Arrows in each substructure of the lungs indicate positive staining of Caveolin. Magnification: ×40; scale bar: 100 µm. The area percentage of Caveolin in control specimens (ctrl) and *yotari* mice per structure (epithelium and mesenchyme) throughout different stages of developing lungs (**e,f**). Two substructures were assessed at E13.5 and E15.5. Data are displayed as the mean ± SD (vertical line) and analyzed by a two-way ANOVA test followed by Tukey’s multiple comparison test. Significant differences are indicated by the following: * *p* < 0.05, *** *p* < 0.001; **** *p* < 0.0001. The statistical details supporting these findings are provided in Supplementary Tables S1 and S2.

Caveolin expression was detected in both epithelial and mesenchymal compartments of the embryonic lung at E13.5 and E15.5 in control and *yotari* mice. The immunofluorescence signal was predominantly cytoplasmic and localized most prominently to the epithelial lining of the developing bronchiolar structures (Figure 3).

At E13.5, the epithelial Caveolin signal was more intense in control lungs compared to *yotari*. This visual observation is supported by the quantification, which revealed a statistically significant reduction in *yotari* at this stage (*p* < 0.05) (Figure 3e). The arrows in Figure 3a,b highlight this difference, pointing to areas of dense epithelial staining in control E13.5, in contrast to the weaker and more diffused signal in *yotari* epithelium.

As development progressed, epithelial Caveolin expression increased markedly in both genotypes between E13.5 and E15.5, with control lungs showing a particularly strong apical localization along the airway epithelium. This developmental upregulation was statistically significant in both groups (*p* < 0.0001 in controls; *p* < 0.001 in *yotari*), but expression remained significantly lower in *yotari* compared to controls at E15.5 (*p* < 0.0001). The arrows at E15.5 indicate these regions of enhanced epithelial staining, showing a continuous and brightly outlined epithelium in control lungs, in contrast to the weaker labeling in *yotari*. These findings highlight a stage-dependent increase in epithelial Caveolin-1 during lung development, which is blunted in *Dab1*-deficient lungs. In contrast, the mesenchymal compartment showed no significant differences in Caveolin expression between any of the groups or timepoints (Figure 3f). The signal within the mesenchyme remained faint and uniform, and quantification confirmed the absence of statistically meaningful variation.

### 3.4. GIPC1 Expression

**Figure 4:**
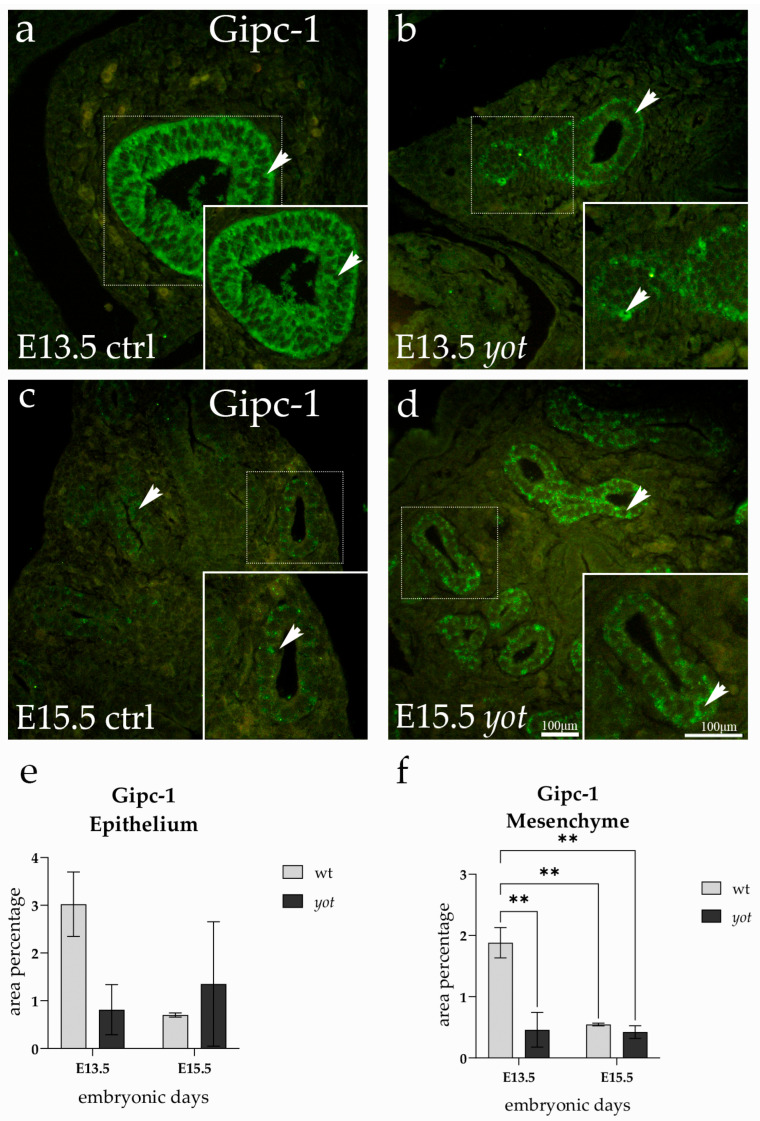
Immunofluorescence staining of Gipc-1 in developing control (ctrl) and *yotari* (yot) lung tissue (**a**–**d**). Comparative expression of Gipc-1 in the lungs at embryonic day 13.5 (E13.5) and 15.5 (E15.5) is shown in (**a**–**d**). Arrows in each substructure of the lungs indicate positive staining of Gipc-1. Magnification: ×40; scale bar: 100 µm. The area percentage of Gipc-1 in control specimens (ctrl) and *yotari* mice per structure (epithelium and mesenchyme) throughout different stages of developing lungs (**e,f**). Two substructures were assessed at E13.5 and E15.5. Data are displayed as the mean ± SD (vertical line) and analyzed by a two-way ANOVA test followed by Tukey’s multiple comparison test. Significant differences are indicated by the following: ** *p* < 0.01. The statistical details supporting these findings are provided in Supplementary Tables S1 and S2.

Gipc-1 expression was detected in both the epithelial and mesenchymal compartments of developing lungs in control and *yotari* embryos at E13.5 and E15.5. The signal was predominantly cytoplasmic and showed a compartment-specific distribution pattern across developmental stages (Figure 4).

Within the epithelium, Gipc-1 exhibited its strongest signal in control lungs at E13.5, with a distinct accumulation along the airway lining, as indicated by arrows (Figure 4a). A similarly prominent signal was also observed in the epithelium of *yotari* lungs at E15.5, although slightly more diffused in appearance (Figure 4b). Despite these visually noticeable differences in intensity, statistical analysis revealed no significant changes in epithelial Gipc-1 levels across any of the experimental groups or developmental stages (Figure 4e). Expression in the epithelium thus remained stable, irrespective of genotype or embryonic age, and the apparent signal variations were not statistically supported.

In contrast, the mesenchymal compartment displayed a more dynamic expression pattern. At E13.5, control lungs showed the highest Gipc-1 signal in the mesenchyme, with numerous positive cells scattered throughout the tissue. This elevated expression was significantly reduced in *yotari* embryos at the same stage (*p* < 0.01), suggesting an early impact of *Dab1* deficiency. An apparent developmental downregulation was also observed, as Gipc-1 expression declined significantly in control lungs from E13.5 to E15.5 (*p* < 0.01). Moreover, *yotari* lungs at E15.5 also exhibited markedly lower mesenchymal expression compared to the high signal seen in control E13.5 lungs (*p* < 0.01) (Figure 4f).

Together, these results indicate that while epithelial Gipc-1 expression remains stable across stages, mesenchymal Gipc-1 undergoes dynamic changes during development, and these changes are accentuated by Dab1 deficiency.

### 3.5. Dab2IP Expression

**Figure 5:**
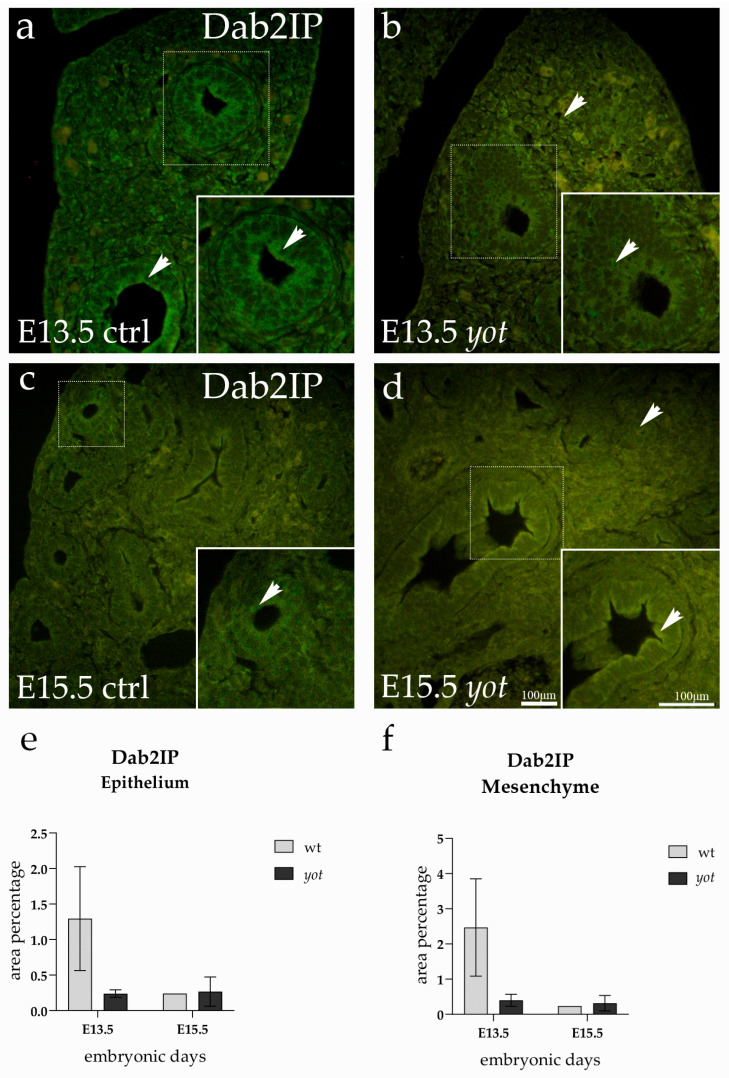
Immunofluorescence staining of Dab2IP in developing control (ctrl) and *yotari* (yot) lung tissue (**a**–**d**). Comparative expression of Dab2IP in the lungs at embryonic day 13.5 (E13.5) and 15.5 (E15.5) is shown in (**a**–**d**). Arrows in each substructure of the lungs indicate positive staining of Dab2IP. Magnification: ×40; scale bar: 100 µm. The area percentage of Dab2IP in control specimens (ctrl) and *yotari* mice per structure (epithelium and mesenchyme) throughout different stages of developing lungs (**e**,**f**). Two substructures were assessed at E13.5 and E15.5. Data are displayed as the mean ± SD (vertical line) and analyzed by a two-way ANOVA test followed by Tukey’s multiple comparison test.

Dab2IP was broadly expressed in both the epithelial and mesenchymal compartments of the developing lung at E13.5 and E15.5 in control and *yotari embryos*. The signal appeared predominantly cytoplasmic, with a soft granular pattern visible across tissue layers (Figure 5).

At E13.5, the epithelium of control lungs displayed the most striking Dab2IP signal, forming a continuous green band along the airway surface, emphasizing the dense accumulation of protein in the epithelial lining (Figure 5a). In addition to the epithelial localization, control lungs at this stage also showed a relatively strong signal within the mesenchyme, where scattered cells exhibited distinct cytoplasmic staining (Figure 5a). In contrast, *yotari* lungs at E13.5 revealed a slightly more diffused and even expression pattern, with less prominent compartmental contrast (Figure 5b).

By E15.5, the overall intensity and distribution of Dab2IP remained relatively stable across all groups. Although subtle shifts in signal localization were visible, particularly in control tissues, quantitative analysis revealed no statistically significant differences between any of the groups or developmental stages (Figure 5e,f). Expression levels in both the epithelium and mesenchyme were consistent across control and *yotari* mice, and no significant changes occurred over time.

## 4. Discussion

Lung development is a highly coordinated process involving complex interactions between epithelial and mesenchymal compartments, governed by a network of morphogens, growth factors, and intracellular signaling pathways [34]. Disruption of these processes can result in developmental anomalies, altered tissue architecture, or impaired respiratory function. Although Dab1 is primarily recognized for its role in neuronal migration via the Reelin signaling cascade, recent studies suggest its involvement in extra-neuronal tissues, including the lung, where it may contribute to epithelial polarity, cytoskeletal organization, and vesicular transport [35]. Our study provides novel insights into the expression profiles of key endocytic and signaling-related proteins, including Megalin, Cubilin, Caveolin-1, GIPC1, and Dab2IP, in the developing lungs of *yotari* mice compared to wild-type controls at embryonic stages E13.5 and E15.5.

Megalin, a large multiligand endocytic receptor of the LDL receptor family, is predominantly expressed in proximal tubules of the kidney but has also been identified in other absorptive epithelia. Its role in the lungs remains mostly unknown. In our study, mesenchymal expression of Megalin was moderately increased in *yotari* mice at E15.5, suggesting a potential compensatory or stress-related response to *Dab1* deficiency. Megalin is known to mediate the endocytosis of various ligands, including albumin, vitamin carriers, and hormones, contributing to tissue homeostasis. In the renal system, Megalin overexpression has been associated with protein overload, lysosomal dysfunction, and inflammation, particularly in models of glomerular injury and diabetic nephropathy [36]. Similar mechanisms may be at play in the developing lung, especially in the context of oxidative stress or abnormal signaling, as previously observed in *yotari* mouse tissues [9,12]. The described mesenchymal upregulation may reflect a need to increase clearance capacity or signal modulation during altered morphogenesis in *Dab1*-deficient lungs. Notably, TGF-β, a critical mediator of lung development and fibrosis, has been shown to downregulate Megalin via GSK3β/PP1 signaling in alveolar epithelial cells [37], linking Megalin expression to growth factor signaling and repair mechanisms. Our findings suggest that Megalin may play a previously underappreciated role in lung morphogenesis, particularly under conditions of disrupted intracellular scaffolding such as *Dab1* loss.

Cubilin, a peripheral membrane protein and co-receptor with Megalin, is essential for the endocytosis of intrinsic factor—vitamin B_12_ complexes and other proteins [38]. While its expression has been extensively studied in the renal proximal tubule and ileal enterocytes, few studies have addressed its role in the lungs. Our data indicate that Cubilin expression increased over time in both genotypes, with slightly elevated levels in *yotari* mice in the epithelial compartment. This temporal pattern suggests a developmentally regulated role for Cubilin in lung maturation, possibly in nutrient uptake, fluid clearance, or surfactant recycling. The interaction between Megalin and Cubilin is well documented in intestinal and renal epithelia [38], where Cubilin relies on Megalin for apical membrane targeting and endocytic function. In our study, the co-expression of these receptors in lung tissue—especially their upregulation in mesenchymal regions—raises the possibility of similar cooperation in pulmonary epithelial transport or alveolar–capillary exchange processes. Importantly, Cubilin expression was relatively unaffected by *Dab1* loss, suggesting its regulation may occur via *Dab1*-independent pathways or be maintained through compensatory signaling. This robustness could reflect its indispensable function during early respiratory epithelial differentiation. As pulmonary epithelial cells undergo structural and functional maturation between E13.5 and E15.5 [2], sustained Cubilin expression may contribute to the establishment of absorptive or clearance functions required for postnatal lung activity.

GIPC1, a PDZ-domain-containing scaffolding protein involved in the trafficking of transmembrane proteins, modulates receptor localization, internalization, and signal strength and has been implicated in cancer progression, angiogenesis, and neuronal development [25]. In our study, GIPC1 expression progressively increased in wild-type lung epithelium and mesenchyme but remained markedly diminished in *yotari* mice, particularly at E15.5. Closer inspection revealed that mesenchymal Gipc-1 expression was dynamically downregulated between E13.5 and E15.5, with *Dab1* deficiency further accentuating this reduction at early stages. At the same time, epithelial levels remained relatively stable despite visually stronger signals at specific timepoints. The dynamic changes in Gipc-1 expression may reflect its role in early branching morphogenesis, consistent with its known interaction with receptors, such as PlexinD1, in guiding tissue patterning [23]. The lack of significant epithelial differences despite visible signal variation suggests that Gipc-1 is more critical in mesenchymal than epithelial compartments during these stages. This indicates that *Dab1* may be essential for the upregulation or stabilization of GIPC1 expression during lung development. Given that GIPC1 modulates the TGFβ signaling [39], which is crucial for mesenchymal proliferation and alveolar formation, its suppression in *yotari* mice may impair cellular responses to different morphogens. Furthermore, impaired GIPC1 expression may compromise receptor trafficking and endocytic sorting, contributing to the developmental delay or epithelial disorganization previously observed in *Dab1*-deficient lungs [12]. This suggests a potential role of *Dab1* in endosomal regulation and morphogen signaling, extending beyond its established functions in neuronal migration.

Dab2IP, an Ras-GAP family member, functions as a tumor suppressor and modulates both the PI3K/AKT and MAPK pathways. It is known to influence epithelial–mesenchymal transition (EMT), apoptosis, and inflammatory signaling [40]. While its role in the lung has been explored in the context of cancer and fibrosis, data on embryonic expression are limited. In our analysis, Dab2IP showed minimal expression in the epithelium of both genotypes; however, a pronounced increase in mesenchymal expression was evident in *yotari* lungs at E15.5, although this difference was not statistically significant. This mesenchymal upregulation may represent a compensatory mechanism in response to *Dab1* deficiency, aiming to restore signaling homeostasis in developing lung tissue. Given its ability to antagonize AKT activation and promote pro-differentiation signals [40], increased Dab2IP may help buffer against aberrant PI3K-mediated proliferation or EMT-like transitions under Dab1-deficient conditions. Alternatively, Dab2IP may participate in establishing epithelial–mesenchymal boundaries during lung branching, a process known to be sensitive to Reelin pathway activity [35]. Our findings support a nuanced interplay between Dab1 and Dab2IP in regulating mesenchymal signaling tone and morphogenetic fidelity during lung development.

Caveolin-1 is a structural protein essential for the formation of caveolae, specialized lipid raft domains involved in vesicular trafficking, mechanotransduction, and signal modulation. It is widely expressed in endothelial and epithelial cells, including alveolar type I cells, and plays key roles in lung development and response to injury [41]. In our study, epithelial Caveolin-1 expression showed a clear developmental increase from E13.5 to E15.5 in control lungs, whereas this upregulation was significantly attenuated in *yotari* mice. In contrast, mesenchymal expression remained low and without significant differences. This stage-dependent epithelial increase likely reflects the mechanosensory role of Caveolin-1, since caveolae act as plasma membrane sensors that buffer mechanical stress and transduce signals related to stretch and shear forces [42]. A rise in epithelial Caveolin-1 during development may therefore represent an adaptive preparation for the mechanical demands of breathing at birth. The blunted increase in *yotari* lungs suggests impaired mechanosensory adaptation, which could limit the ability of epithelial cells to appropriately respond to growth factor signaling and mechanical stimuli during branching morphogenesis and alveolar maturation. In addition, Caveolin-1 has been implicated in lung fibrosis and remodeling, with earlier studies showing its role as a regulator of tissue injury and repair [43], and more recent work confirming its critical functions in maintaining pulmonary homeostasis [20]. Caveolin-1 is known to influence TGF-βsignaling, nitric oxide homeostasis, and actin cytoskeletal dynamics, all of which are critical for alveolar maturation and airway stabilization [42]. Its upregulation in *Dab1*-deficient epithelium may thus reflect enhanced cellular stress, altered mechanosensing, or increased need for membrane compartmentalization. Notably, previous studies have linked Caveolin-1 to lung fibrosis and impaired repair in chronic lung diseases, emphasizing its dual role in both development and disease [44]. The high Caveolin-1 levels observed in *yotari* lungs may signal disrupted cytoskeletal dynamics or morphogen gradient interpretation, potentially contributing to structural abnormalities or developmental delays. Taken together, our findings suggest a link between *Dab1* deficiency, impaired epithelial mechanosensory regulation, and abnormal structural development, thereby extending the role of *Dab1* signaling into the domain of epithelial mechanobiology.

Limitations of the present study should be acknowledged. The sample size was limited to three embryos per genotype and developmental stage, which, although standard in embryological investigations, constrains statistical power and may not fully capture the range of biological variability. In addition, the study relied on immunohistochemical analysis of protein expression, which provides spatial resolution but does not directly address functional consequences. Future studies should aim to dissect the downstream pathways through which *Dab1* modulates these targets and evaluate the functional consequences of their dysregulation in lung morphogenesis and disease susceptibility. Other complementary techniques, such as Western blotting or RNA-based approaches, could strengthen future analyses.

## 5. Conclusions

This study identifies *Dab1* as a broader regulator of lung development than previously recognized. By altering the expression of key proteins involved in endocytosis, signal transduction, and cytoskeletal organization, *Dab1* deficiency disrupts epithelial–mesenchymal crosstalk and intracellular transport dynamics essential for proper morphogenesis. The observed changes in Megalin, Cubilin, GIPC1, Dab2IP, and Caveolin-1 highlight *Dab1*’s role as a potential central coordinator of endocytic receptor expression in non-neuronal tissues during periods of rapid growth and remodeling. Together, our findings provide new insight into the molecular mechanisms of lung organogenesis and suggest that *Dab1* may represent a critical node linking developmental signaling pathways with structural maturation.

## Figures and Tables

**Table 1:** Antibodies used for immunofluorescence.

Antibodies		Host	Dilution	Source
Primary	Anti-Lrp2/Megalin antibody	Rabbit	1:250	Abcam, Cambridge, UK
	Human/Mouse/Rat Cubilin Antibody (#AF3700)	Sheep	1:50	R&D Systems, Inc., Minneapolis, MN, USA
	Caveolin-1 (D46G3) XP^®^ Rabbit mAb (#3267S)	Rabbit	1:300	Cell Signaling Technology (CST), Danvers, MA, USA
	Gipc1 Polyclonal antibody (14822-1-AP)	Rabbit	1:100	Proteintech Group, Inc., Rosemont, IL, USA
	Dab2IP Polyclonal antibody (23582-1-AP)	Rabbit	1:50	Proteintech Group, Inc., Rosemont, IL, USA
Secondary	Alexa Fluor^®^ 488 AffiniPure^TM^ Donkey Anti-Rabbit IgG (H + L) (711-545-152)	Donkey	1:300	Jackson Immuno Research Laboratories, Inc., West Groove, PA, USA
	Rhodamine Red^TM^-X (RRX) AffiniPure^TM^ Donkey Anti-Rabbit IgG (H + L) (711-295-152)	Donkey	1:300	Jackson Immuno Research Laboratories, Inc., West Groove, PA, USA

## Data Availability

All data and materials are available upon request.

## Supplementary Materials

The following supporting information can be downloaded at: https://www.mdpi.com/article/10.3390/life15091395/s1, Table S1: Two-way ANOVA summary of protein expression in developing control (ctrl) and *Dab1*-deficient (*yotari*) lungs; Table S2: Tukey’s multiple comparisons test for protein expression across developmental stages (E13.5, E15.5) in control (ctrl) and *yotari* (*yot*) lungs.
