# Supplementary material for: Loss of Dab1 Alters Expression Patterns of Endocytic and Signaling Molecules During Embryonic Lung Development in Mice
Source: Life (Basel). 2025 Sep 3;15(9):1395. doi: 10.3390/life15091395 (PMC12471013; doi:10.3390/life15091395)
Supplement: Supplementary file 1 [file life-15-01395-s001.zip › life-3824172-supplementary.pdf]

Supplementary Table S1. Two-way ANOVA summary of protein expression in developing control (ctrl) and *Dab1*-deficient (*yotari*) lungs.

Protein	Compartment	Source of Variation	F (DFn, DFd)	p-value	Significance
**Megalin**	Epithelium	Interaction	F(1,12)=0.878	0.3673	ns
		Genotype (ctrl vs. *yot*)	F(1,12)=1.528	0.2401	ns
		Stage (E13.5 vs. E15.5)	F(1,12)=0.020	0.8886	ns
	Mesenchyme	Interaction	F(1,12)=0.0285	0.8686	ns
		Genotype	F(1,12)=16.38	0.0016	**
		Stage	F(1,12)=0.619	0.4465	ns
**Cubilin**	Epithelium	Interaction	F(1,11)=0.0884	0.7718	ns
		Genotype	F(1,11)=0.264	0.6177	ns
		Stage	F(1,11)=4.195	0.0652	ns
	Mesenchyme	Interaction	F(1,11)=0.0116	0.9163	ns
		Genotype	F(1,11)=1.238	0.2895	ns
		Stage	F(1,11)=3.787	0.0777	ns
**Caveolin-1**	Epithelium	Interaction	F(1,3)=1233	<0.0001	****
		Genotype	F(1,3)=1833	<0.0001	****
		Stage	F(1,3)=5433	<0.0001	****
	Mesenchyme	Interaction	F(1,3)=1.696	0.2837	ns
		Genotype	F(1,3)=2.014	0.2510	ns
		Stage	F(1,3)=3.093	0.1768	ns
**GIPC1**	Epithelium	Interaction	F(1,4)=6.739	0.0603	ns
		Genotype	F(1,4)=2.008	0.2294	ns
		Stage	F(1,4)=2.612	0.1813	ns
	Mesenchyme	Interaction	F(1,4)=21.94	0.0094	**
		Genotype	F(1,4)=31.16	0.0050	**
		Stage	F(1,4)=24.52	0.0077	**
**Dab2IP**	Epithelium	Interaction	F(1,6)=1.464	0.2718	ns
		Genotype	F(1,6)=1.320	0.2943	ns
		Stage	F(1,6)=1.311	0.2958	ns
	Mesenchyme	Interaction	F(1,6)=1.636	0.2481	ns
		Genotype	F(1,6)=1.893	0.2180	ns
		Stage	F(1,6)=1.400	0.2814	ns

Statistical analysis was performed using two-way ANOVA to assess the effects of genotype control vs. *yotari* (ctrl vs. *yot*), developmental stage (embryonic day E13.5 vs. E15.5), and their interaction on the expression of Megalin, Cubilin, Caveolin-1, GIPC1, and Dab2IP in epithelial and mesenchymal compartments of embryonic lungs. The table reports the F values, degrees of freedom (DFn, DFd), p-values, and levels of statistical significance for each

factor. Significance thresholds were set as follows: $p < 0.05$ (*), $p < 0.01$ (**), $p < 0.001$ (***), $p < 0.0001$ (****), and "ns" = not significant.

Supplementary Table S2. Tukey's multiple comparisons test for protein expression across developmental stages (E13.5, E15.5) in control (ctrl) and *yotari* (*yot*) lungs.

Protein	Compartment	Comparison	Mean diff.	95% CI of diff.	Significance	Adjusted P value
**Megalin**	Epithelium	E13.5 ctrl vs. E13.5 *yot*	0.041	–0.437 to 0.520	ns	>0.9999
		E13.5 ctrl vs. E15.5 ctrl	–0.149	–0.810 to 0.512	ns	0.9830
		E13.5 *yot* vs. E15.5 *yot*	0.109	–0.449 to 0.668	ns	0.9917
		E15.5 ctrl vs. E15.5 *yot*	0.299	–0.422 to 1.020	ns	0.7692
	Mesenchyme	E13.5 ctrl vs. E13.5 *yot*	0.697	0.154 to 1.240	*	0.0114
		E13.5 ctrl vs. E15.5 ctrl	0.158	–0.592 to 0.908	ns	0.9218
		E13.5 *yot* vs. E15.5 *yot*	0.102	–0.532 to 0.736	ns	0.9624
		E15.5 ctrl vs. E15.5 *yot*	0.641	–0.177 to 1.459	ns	0.1462
**Cubilin**	Epithelium	E13.5 ctrl vs. E13.5 *yot*	0.781	–2.566 to 4.128	ns	0.8941
		E13.5 ctrl vs. E15.5 ctrl	–1.686	–6.177 to 2.804	ns	0.6795
		E13.5 *yot* vs. E15.5 *yot*	–2.259	–5.925 to 1.407	ns	0.3008
		E15.5 ctrl vs. E15.5 *yot*	0.208	–4.525 to 4.942	ns	0.9991
	Mesenchyme	E13.5 ctrl vs. E13.5 *yot*	1.952	–1.351 to 5.254	ns	0.3330
		E13.5 ctrl vs. E15.5 ctrl	–0.956	–5.387 to 3.476	ns	0.9137
		E13.5 *yot* vs. E15.5 *yot*	–1.160	–4.778 to 2.458	ns	0.7716

Protein	Compartment	Comparison	Mean diff.	95% CI of diff.	Significance	Adjusted P value
		E15.5 ctrl vs. E15.5 *yot*	1.747	–2.924 to 6.418	ns	0.6821
**Caveolin**	Epithelium	E13.5 ctrl vs. E13.5 *yot*	0.053	0.017 to 0.090	*	0.0173
		E13.5 ctrl vs. E15.5 ctrl	–0.755	–0.801 to –0.709	****	<0.0001
		E13.5 *yot* vs. E15.5 *yot*	–0.268	–0.316 to –0.219	***	0.0004
		E15.5 ctrl vs. E15.5 *yot*	0.540	0.484 to 0.597	****	<0.0001
	Mesenchyme	E13.5 ctrl vs. E13.5 *yot*	1.921	–1.364 to 5.206	ns	0.1835
		E13.5 ctrl vs. E15.5 ctrl	1.708	–2.447 to 5.862	ns	0.3585
		E13.5 *yot* vs. E15.5 *yot*	0.073	–4.334 to 4.480	ns	0.9998
		E15.5 ctrl vs. E15.5 *yot*	0.286	–4.802 to 5.375	ns	0.9916
**GIPC1**	Epithelium	E13.5 ctrl vs. E13.5 *yot*	2.211	–0.961 to 5.384	ns	0.1445
		E13.5 ctrl vs. E15.5 ctrl	2.321	–0.851 to 5.494	ns	0.1269
		E13.5 *yot* vs. E15.5 *yot*	–0.540	–3.712 to 2.633	ns	0.8950
		E15.5 ctrl vs. E15.5 *yot*	–0.650	–3.822 to 2.523	ns	0.8371
	Mesenchyme	E13.5 ctrl vs. E13.5 *yot*	1.421	0.624 to 2.217	**	0.0066
		E13.5 ctrl vs. E15.5 ctrl	1.333	0.537 to 2.130	**	0.0083
		E13.5 *yot* vs. E15.5 *yot*	0.037	–0.760 to 0.834	ns	0.9972
		E15.5 ctrl vs. E15.5 *yot*	0.124	–0.672 to 0.921	ns	0.9156
**DAB2IP**	Epithelium	E13.5 ctrl vs. E13.5 *yot*	1.055	–0.692 to 2.803	ns	0.2562
		E13.5 ctrl vs. E15.5 ctrl	1.054	–1.234 to 3.341	ns	0.4466
		E13.5 *yot* vs. E15.5 *yot*	–0.029	–2.117 to 2.059	ns	>0.9999

Protein	Compartment	Comparison	Mean diff.	95% CI of diff.	Significance	Adjusted P value
		E15.5 ctrl vs. E15.5 *yot*	–0.027	–2.585 to 2.530	ns	>0.9999
	Mesenchyme	E13.5 ctrl vs. E13.5 *yot*	2.072	–1.214 to 5.358	ns	0.2300
		E13.5 ctrl vs. E15.5 ctrl	2.234	–2.069 to 6.537	ns	0.3588
		E13.5 *yot* vs. E15.5 *yot*	0.082	–3.846 to 4.009	ns	0.9999
		E15.5 ctrl vs. E15.5 *yot*	–0.080	–4.891 to 4.730	ns	>0.9999

Tukey's multiple comparisons test for protein expression (Megalin, Cubilin, Caveolin-1, GIPC1, and DAB2IP) in the epithelium and mesenchyme compartments of embryonic lungs at E13.5 and E15.5 in control (ctrl) and *yotari* (*yot*) mice. The table lists all pairwise comparisons between groups, with mean difference, 95% confidence interval (CI), adjusted p-values, and significance levels. Significance is indicated as follows: $p < 0.05$ (*), $p < 0.01$ (**), $p < 0.001$ (***), $p < 0.0001$ (****), *and "ns" = not significant.*
